# School-age somatic, internalizing, attention, and social difficulties in moderate anthropometric undernutrition: a primary-care case-control study

**DOI:** 10.3389/fped.2026.1893524

**Published:** 2026-08-18

**Authors:** Dilek Altun Varmış, Selda Pamiry

**Affiliations:** 1 Department of Child and Adolescent Psychiatry, University of Health Sciences Adana City Training and Research Hospital, Adana, Türkiye; 2 Seyhan Gülbahçesi Family Health Center, Adana, Türkiye

**Keywords:** anthropometric undernutrition, attention problems, child mental health, internalizing symptoms, moderate wasting, primary care, somatic complaints, thinness

## Abstract

**Background:**

Moderate anthropometric undernutrition is associated with developmental risks, but the emotional, behavioral, and functional profiles of children with moderate anthropometric undernutrition managed in outpatient primary care follow-up remain insufficiently characterized.

**Methods:**

This cross-sectional case-control study examined 60 mother-child dyads in Adana, Türkiye (undernutrition, *n* = 30; control, *n* = 30). Children were assessed by age group using the Child Behavior Checklist, and mothers completed parent-proxy measures of quality of life, psychological distress, and parenting-related emotion regulation.

**Results:**

No statistically significant between-group differences were detected in the measured sociodemographic and general clinical variables, although the sample was not powered to establish equivalence or exclude residual confounding. Children with moderate anthropometric undernutrition were more often described as eating less or showing selective eating, and family relationship quality was less often rated as good. Parent-proxy quality of life, maternal psychological distress, and parenting-related emotion regulation did not differ at the group level. Preschool CBCL scores were comparable. In the CBCL/6-18 subgroup, primary comparisons using age- and sex-normed *T*-scores identified higher Somatic Complaints, Anxious/Depressed, Social Problems, Attention Problems, Internalizing Problems, and Total Problems scores in the undernutrition group. Somatic Complaints (*q* = 0.020), Attention Problems (*q* = 0.036), Anxious/Depressed (*q* = 0.047), and Internalizing Problems (*q* = 0.047) remained significant after false-discovery-rate correction; Social Problems and Total Problems did not survive correction and are interpreted as preliminary. Externalizing Problems were not elevated at the group level. Within the undernutrition group, higher internalizing and externalizing *T*-scores were associated with poorer parent-proxy quality of life in the primary Pearson analyses, although these associations were attenuated and did not survive correction in the all-Spearman sensitivity analysis.

**Conclusion:**

Because of the cross-sectional design, the direction of these associations cannot be established; they may reflect nutritional influences on psychosocial functioning, behavioral or feeding difficulties contributing to low weight, or shared antecedents of both. These findings suggest a selective school-age psychosocial screening signal, rather than generalized impairment, in outpatient moderate anthropometric undernutrition. Routine nutrition follow-up may benefit from brief behavioral screening, feeding behavior assessment, and family-sensitive primary care support.

## Introduction

Undernutrition remains a major pediatric public health concern because it is associated with morbidity, mortality, impaired development, and losses in human capital (1). Contemporary child nutrition challenges are increasingly framed within the triple burden of malnutrition, which includes undernutrition, micronutrient deficiency, and overweight or obesity (2). Global estimates indicate that chronic undernutrition, reflected by stunting, continues to affect many children under 5 years of age (3). Burden-of-disease and social-determinants research further shows that malnutrition is distributed unequally across sociodemographic contexts (4, 5). Pediatric evidence also indicates that undernutrition is embedded within clinical and social circumstances rather than being an isolated anthropometric state (6).

Nutrition is central to neurodevelopment and cognitive functioning, with evidence linking early nutrient availability to brain maturation and cognitive development (7, 8). Evidence from severe acute malnutrition, childhood stunting, and systematic reviews indicates that childhood malnutrition may be associated with cognitive, neurodevelopmental, behavioral, and mental-health outcomes (9–12). However, these studies do not fully answer a practical primary-care question: whether children with moderate anthropometric undernutrition who are managed as outpatients show detectable emotional, behavioral, or quality-of-life difficulties during routine follow-up. In the present study, moderate anthropometric undernutrition encompasses age-appropriate WHO categories of moderate wasting in children younger than 5 years and thinness in children aged 5 years or older.

This gap is important for child and adolescent mental health. Moderate undernutrition may be less visible than severe acute malnutrition and may be monitored mainly through growth indicators. Yet developmental psychopathology models suggest that child outcomes emerge through interactions among biological vulnerability, caregiving responses, and environmental demands. Transactional developmental theory emphasizes bidirectional processes between child functioning and caregiving contexts over time (13). In the present cross-sectional study, this framework was used as a developmental interpretive background rather than as a longitudinal causal model to be tested. Parenting-related emotion regulation is also clinically relevant because caregivers regulate both their own emotional responses and their responses to children's emotional signals (14, 15). An outpatient undernutrition follow-up model that focuses only on physical growth may therefore miss mental health, feeding-context, and family-process signals that become more visible when children enter school, face higher attentional demands, or navigate more complex peer contexts.

The present study examined behavioral and emotional symptoms, parent-proxy health-related quality of life, feeding-related characteristics, maternal psychological distress, and parenting-related emotion regulation in children with clinically documented moderate anthropometric undernutrition followed in a primary-care setting in Türkiye. The primary objective was to compare age-stratified behavioral and emotional profiles between children with moderate anthropometric undernutrition and healthy controls using developmentally appropriate CBCL forms. The secondary objectives were to compare parent-proxy health-related quality of life, maternal psychological distress, parenting-related emotion regulation, and feeding- and family-context characteristics between groups. The exploratory objective was to examine whether child symptoms and maternal variables were associated with perceived child quality of life within each group. We expected that school-age children with moderate anthropometric undernutrition would show a more detectable emotional and behavioral profile than preschool children because school-age contexts increase demands on attention, self-regulation, and social functioning. By focusing on an outpatient primary-care population, the study aimed to inform follow-up models that consider nutritional status alongside child mental health, feeding behavior, family processes, and functional well-being.

## Materials and methods

### Study design and setting

This cross-sectional case-control study was conducted at the Gülbahçesi Family Health Center in Adana, Türkiye. The study compared children and adolescents with clinically documented moderate anthropometric undernutrition under ongoing outpatient nutritional follow-up with healthy controls from the same community catchment area who were selected to be broadly comparable by developmental assessment band where feasible. The undernutrition diagnosis and anthropometric classification were established through tertiary-care pediatric outpatient services, and ongoing monitoring took place in primary care in line with routine child and adolescent follow-up protocols for nutritional rehabilitation in Türkiye (16).

### Participants and sampling

The analytic sample included 60 child-mother dyads: 30 children with moderate anthropometric undernutrition and 30 controls. Participants were recruited between February 15 and April 15, 2026. Children in the undernutrition group were identified from those under ongoing outpatient nutritional follow-up at the Gülbahçesi Family Health Center after tertiary-care pediatric outpatient diagnosis of moderate anthropometric undernutrition. Eligible undernutrition cases who attended routine follow-up visits during the recruitment period were approached consecutively. Case eligibility was verified through medical records, anthropometric measurements, and documentation of ongoing outpatient nutritional management.

Controls were recruited during the same timeframe from children and adolescents attending the same family health center for routine preventive, growth-monitoring, or non-malnutrition-related primary-care visits. Control participants were screened to ensure growth parameters within normal limits, no previous diagnosis of malnutrition, and no chronic medical condition affecting growth or development. Controls were enrolled using convenience sampling from eligible healthy children who met these screening criteria and attended the same family health center during the recruitment period; they were selected to be as similar as possible in age and developmental assessment band to children in the undernutrition group where feasible, and the same general inclusion and exclusion criteria were applied to both groups except for undernutrition status.

The mean child age was 7.87 years (SD = 3.48), and 34 children (56.7%) were girls. All questionnaires were completed in Turkish by biological mothers who were the primary caregivers. Participants were stratified into two developmental assessment bands to match standardized CBCL forms: preschool children aged 1.5–5 years and school-age participants aged 6–18 years. The school-age label is used for brevity and refers to the CBCL/6-18 assessment band, which includes adolescents. These assessment bands were used only for age-appropriate measurement and do not imply longitudinal follow-up or observed developmental transitions.

### Sample-size justification

The sample size was determined by the number of eligible children with clinically documented moderate anthropometric undernutrition who were under outpatient primary-care follow-up during the predefined two-month recruitment period and by recruitment of an equal number of controls from the same community catchment area. A formal *a priori* power calculation was not performed because the study was designed as an exploratory primary-care case-control study in an undercharacterized outpatient clinical group. With 30 participants per group, the study had approximately 80% power to detect a moderate-to-large between-group standardized mean difference of approximately 0.74 at a two-sided alpha level of 0.05. Therefore, the study was not powered to detect small effects, conduct multivariable modeling, or provide definitive subgroup estimates. The findings should accordingly be interpreted as exploratory screening signals requiring confirmation in larger longitudinal samples.

### Case definition and anthropometric assessment

Anthropometric undernutrition was classified using age-appropriate World Health Organization (WHO) indicators and references (17, 18). *Z*-scores were used for case classification and eligibility and were not treated as outcome variables in the psychosocial comparisons. Among children younger than 5 years, moderate wasting was defined as a weight-for-height *z*-score from −3 SD to <−2 SD according to the WHO Child Growth Standards. Among children aged 5 years or older, thinness without severe thinness was defined as a BMI-for-age *z*-score from −3 SD to <−2 SD according to the WHO 2007 Growth Reference. Because the sample spanned both age groups, these age-specific categories are collectively referred to as moderate anthropometric undernutrition in this study. Children with z-scores from −2 SD to <−1 SD were not included in the undernutrition group, so the analytic sample did not combine children who were only at risk of undernutrition with those meeting the prespecified anthropometric criterion. This restriction was used to minimize measurement heterogeneity. Participants in the undernutrition group did not require hospitalization and were receiving outpatient nutritional management as part of routine clinical follow-up. The exact interval from initial tertiary-care diagnosis to study assessment was not uniformly available in days in the primary-care records; therefore, enrollment was defined by active outpatient nutritional follow-up and continued fulfillment of the age-appropriate anthropometric criterion at study assessment. Nutritional management details were used for case verification but were not analyzed as between-group variables because outpatient nutritional management formed part of the undernutrition case definition. Children in the undernutrition group were assessed while they continued to meet the relevant anthropometric criterion, without anthropometric recovery at study assessment. Children with severe wasting or severe thinness, nutritional edema, or complications requiring hospitalization were excluded. Controls had growth parameters within normal limits (−2 SD ≤ *z*-score ≤ +2 SD), no prior undernutrition diagnosis, and no chronic condition affecting growth or development. Weight was measured to the nearest 0.1 kg using a digital scale, and height or length was measured to the nearest 0.1 cm using a stadiometer or infantometer by trained research personnel.

### Eligibility criteria

Inclusion criteria were child age 1.5–18 years, biological mother available to complete Turkish-language assessments, documented moderate anthropometric undernutrition with ongoing outpatient nutritional management for the undernutrition group, and written informed consent. Exclusion criteria for both groups were known neurological disorder or genetic syndrome, severe intellectual disability, acute febrile illness at the time of assessment, and sensory or communication impairment that prevented valid caregiver report. Efforts to limit bias included recruitment of cases and controls from the same catchment population and clinical service, standardized eligibility criteria, use of age-appropriate normed instruments, and a single standardized assessment session for all participants.

### Measures

A researcher-developed sociodemographic and clinical form collected child demographics, school attendance, academic performance, parental education and occupation, socioeconomic indicators, medical history, breastfeeding history, feeding behavior, weight change during the previous 6 months, peer relationship quality, and caregiver-rated family relationship quality. Nutritional management information was used only to verify undernutrition case status and was not analyzed as a between-group exposure.

Child behavioral and emotional problems were assessed using age-appropriate CBCL forms from the Achenbach System of Empirically Based Assessment. The CBCL/1.5-5 and CBCL/6-18 are caregiver-report instruments that rate symptoms on a 3-point scale, with higher scores indicating greater difficulties (19, 20). The CBCL/1.5-5 covers the previous 2 months, and the CBCL/6-18 covers the previous 6 months. The Turkish CBCL forms have been adapted and validated in Turkish samples (21, 22). For the CBCL/6-18 subgroup, raw syndrome and broadband scores were converted to age- and sex-normed *T*-scores using the licensed ASEBA profiles for girls and boys and the applicable 6–11- or 12–18-year age band. Primary CBCL/6-18 group comparisons and CBCL-PedsQL correlations used these *T*-scores. In accordance with ASEBA interpretation, syndrome-scale *T*-scores of 65–69 were considered borderline and scores of 70 or higher clinical; for Internalizing Problems, Externalizing Problems, and Total Problems, *T*-scores of 60–63 were considered borderline and scores of 64 or higher clinical. Clinical-range prevalence was not treated as an outcome because the study was designed for continuous between-group comparisons. CBCL/1.5-5 analyses retained the available raw scores and were conducted separately.

Child health-related quality of life was assessed using the PedsQL 4.0 Generic Core Scales, Parent Proxy Report. The PedsQL assesses Physical, Emotional, Social, and School Functioning, with reverse-scored items transformed to a 0–100 scale in which higher scores indicate better quality of life (23). Turkish validation studies support the use of age-appropriate parent-proxy forms in pediatric samples (24–26).

Maternal psychological distress was assessed with the 53-item BSI, which measures psychological symptoms during the previous week on a 5-point Likert scale (27). The Turkish adaptation yields symptom dimensions including somatization, depression, anxiety, hostility, and negative self-concept, as well as global indices such as the Global Severity Index (28).

Parenting-related emotion regulation was assessed using the Turkish short form of the PERS, an instrument originally developed for emotion regulation in the parenting context and adapted into Turkish (14, 29). The Turkish short form includes orientation to the child's emotions, avoidance of the child's emotions, and acceptance of the child's and parent's own emotions. Higher scores indicate greater endorsement of corresponding parenting-related emotion-regulation patterns or difficulties.

Internal consistency coefficients were calculated for the principal scale scores used in the present analyses. Cronbach's alpha values in the current sample were acceptable to excellent: CBCL Total Problems score, *α* = 0.89; PedsQL Total Score, *α* = 0.84; BSI Global Severity Index, *α* = 0.92; and PERS total score, *α* = 0.80.

### Procedure

During the aforementioned clinical visits, mothers received detailed information about the study and provided written informed consent. Questionnaires were completed in a single session of approximately 40–50 min in a private room. Trained research personnel were available to clarify instructions without influencing responses. Completed forms were reviewed before participants left the clinic, and missing items were addressed immediately to maximize data completeness. For the undernutrition group, diagnostic classification, outpatient nutritional management verification, and growth-monitoring data were extracted from medical records.

### Statistical analysis

Analyses were conducted using IBM SPSS Statistics version 25.0. Categorical variables were summarized as frequencies and percentages; continuous variables were summarized as means and standard deviations or medians and ranges according to distributional characteristics. Normality was evaluated using Shapiro–Wilk tests and visual inspection. Group comparisons used chi-square or Fisher exact tests for categorical variables and independent samples t-tests or Mann–Whitney U tests for continuous variables, as appropriate. For selected categorical feeding and family-context indicators, unadjusted odds ratios (ORs) with 95% confidence intervals (CIs) were calculated from 2 × 2 contrasts. Continuous questionnaire outcomes were retained on their original continuous scales and were not dichotomized. CBCL analyses were conducted separately for the CBCL/1.5-5 and CBCL/6-18 assessment groups. Age- and sex-normed T-scores constituted the primary metric for all CBCL/6-18 between-group comparisons and CBCL-PedsQL correlations; available raw CBCL/1.5-5 scores were analyzed separately. Pearson correlations were used for the prespecified CBCL-PedsQL analyses to quantify linear associations, whereas Spearman rank correlations were used for maternal BSI and PERS analyses because of their distributional characteristics. Given the skewness of the CBCL and PedsQL distributions and the small CBCL/6-18 subgroup, all 16 within-group correlations were additionally re-evaluated using Spearman rank correlations as a distributional sensitivity analysis, with *T*-scores retained for the CBCL/6-18 variables. Benjamini-Hochberg correction was applied separately to the 16 primary correlation *p*-values and to the 16 Spearman sensitivity *p*-values. Statistical significance was set at a two-tailed *p*-value < 0.05. To aid interpretation of between-group differences, Hedges’ g was calculated from *T*-scores using the pooled standard deviation and small-sample correction; positive values indicate higher scores in the undernutrition group. Ninety-five percent CIs were obtained using a noncentral-t-based method. Effect sizes were interpreted descriptively and not as confirmatory tests. As a secondary robustness analysis, the former raw-score comparisons were retained and repeated using ANCOVA with child age and sex as covariates, rank-transformed ANCOVA, and age- and sex-residualized Hedges' g estimates (Supplementary Table S1). These within-sample models were not used in place of normative standardization and do not exclude residual confounding. Additional multivariable adjustment was not undertaken because the CBCL/6-18 subgroup included only 43 participants and models incorporating several potentially correlated sociodemographic and family variables would have been prone to overfitting and unstable estimation. Given the number of comparisons, the false discovery rate was controlled using the Benjamini-Hochberg procedure within instrument- and domain-based test families: CBCL/6-18, CBCL/1.5-5, PedsQL, BSI, PERS, feeding and family-context indicators, and within-group correlations. The FDR threshold was *q* < 0.05; nominal findings not meeting this threshold were interpreted as preliminary. Adjusted q-values are reported in Supplementary Table S2. Because the CBCL/6-18 *T*-score distributions showed substantial floor-related ties and departures from normality, between-group comparisons were performed using two-sided Mann–Whitney U tests. Hedges' g with 95% CIs was reported as a complementary standardized mean-difference measure. Missing data were not imputed; analyses used all available observations within each instrument and age form, and instrument-specific denominators are reported in the tables.

### Ethics

The study was conducted in accordance with internationally accepted ethical principles for research involving human participants. Ethical approval was granted by the Scientific Research Ethics Committee of Adana City Training and Research Hospital (Meeting No. 19, Decision No. 851, November 20, 2025). Written informed consent was obtained from all participating mothers before data collection.

## Results

### Participant demographic characteristics

The final sample included 60 mother-child dyads: 30 children with clinically documented moderate anthropometric undernutrition under ongoing outpatient nutritional follow-up and 30 healthy controls. Overall, 34 children were girls (56.7%) and 26 were boys (43.3%). The mean child age was 7.87 years (SD = 3.48). The mean maternal age was 33.08 years (SD = 7.28), and the mean paternal age was 37.07 years (SD = 7.82). As shown in Table 1, the undernutrition and control groups did not differ significantly in sex distribution, school attendance, academic performance among school-attending children, parental education, socioeconomic status, parental union, family chronic disease history, child past illness, child chronic disease, medication use, or peer relationship quality (all *p* > 0.05).

**Table 1:** Sociodemographic and clinical characteristics by group.

Variable	Undernutrition *n* (%)	Control *n* (%)	*p*
Sex: female	14 (46.7)	20 (66.7)	0.118
Sex: male	16 (53.3)	10 (33.3)	
School attendance: not attending	8 (26.7)	6 (20.0)	0.566
School attendance: attending	22 (73.3)	24 (80.0)	
Academic performance: good	13 (56.5)	13 (52.0)	0.933
Academic performance: average	8 (34.8)	10 (40.0)	
Academic performance: low	2 (8.7)	2 (8.0)	
Maternal education: illiterate	7 (23.3)	5 (16.7)	0.180
Maternal education: primary school	10 (33.3)	8 (26.7)	
Maternal education: middle school	11 (36.7)	8 (26.7)	
Maternal education: high school	2 (6.7)	8 (26.7)	
Maternal education: university	0 (0.0)	1 (3.3)	
Paternal education: illiterate	8 (26.7)	2 (6.7)	0.265
Paternal education: primary school	9 (30.0)	9 (30.0)	
Paternal education: middle school	6 (20.0)	9 (30.0)	
Paternal education: high school	5 (16.7)	6 (20.0)	
Paternal education: university	2 (6.7)	4 (13.3)	
Socioeconomic status: low	13 (43.3)	17 (56.7)	0.302
Socioeconomic status: middle	17 (56.7)	13 (43.3)	
Family chronic disease history: no	26 (86.7)	26 (86.7)	>0.999
Family chronic disease history: yes	4 (13.3)	4 (13.3)	
Child past illness: no	30 (100.0)	29 (96.7)	>0.999
Child past illness: yes	0 (0.0)	1 (3.3)	
Child chronic disease: no	29 (96.7)	30 (100.0)	>0.999
Child chronic disease: yes	1 (3.3)	0 (0.0)	
Regular medication use: no	29 (96.7)	30 (100.0)	>0.999
Regular medication use: yes	1 (3.3)	0 (0.0)	
Eating behavior: normal	1 (3.3)	17 (56.7)	<0.001
Eating behavior: eats less	15 (50.0)	6 (20.0)	
Eating behavior: overeats	1 (3.3)	0 (0.0)	
Eating behavior: selective eating	13 (43.3)	7 (23.3)	
Weight change in last 6 months: no	18 (60.0)	29 (96.7)	0.001
Weight change in last 6 months: yes	12 (40.0)	1 (3.3)	
Family relationship quality: good	19 (63.3)	27 (90.0)	0.030
Family relationship quality: moderate	11 (36.7)	3 (10.0)	
Parental union: married	30 (100.0)	29 (96.7)	>0.999
Parental union: bereavement	0 (0.0)	1 (3.3)	
Peer relationships: good	24 (80.0)	25 (83.3)	0.936
Peer relationships: moderate	5 (16.7)	4 (13.3)	
Peer relationships: problematic	1 (3.3)	1 (3.3)	

Data are presented as *n* (%). *P*-values were obtained using chi-square or Fisher exact tests, as appropriate. Academic performance data were available only for school-attending children. Nutritional management was part of the Undernutrition case definition and was not analyzed as a between-group characteristic. Undernutrition, moderate anthropometric undernutrition.

### Feeding and family context

As expected by case definition, children in the undernutrition group were under ongoing outpatient nutritional management and continued to meet moderate anthropometric criteria at assessment. Because nutritional management was part of the undernutrition case definition, it was not analyzed as a between-group characteristic. Recent weight change in the previous 6 months was more frequent in the undernutrition group than in controls (40.0% vs. 3.3%, *p* = 0.001). Eating behavior also differed between groups (overall comparison, *p* < 0.001), with eating less and selective eating more common in the undernutrition group. Mothers in the undernutrition group were less likely to endorse overall family relationship quality as good (63.3% vs. 90.0%, *p* = 0.030). A summary of unadjusted ORs with 95% CIs for these selected categorical indicators is provided in Table 2. After Benjamini-Hochberg correction across the five feeding and family-context indicators, non-normal eating behavior (*q* < 0.001), weight change during the previous six months (*q* = 0.003), eating less (*q* = 0.038), and family relationship quality not rated as good (*q* = 0.038) remained statistically significant. Selective eating did not remain significant (*q* = 0.170).

**Table 2:** Unadjusted odds ratios for selected categorical feeding and family-context indicators.

Indicator	Undernutrition, n/N (%)	Control, n/N (%)	Unadjusted OR (95% CI)	*p*
Non-normal eating behavior	29/30 (96.7)	13/30 (43.3)	37.92 (4.55–316.03)	<0.001
Eats less	15/30 (50.0)	6/30 (20.0)	4.00 (1.27–12.58)	0.029
Selective eating	13/30 (43.3)	7/30 (23.3)	2.51 (0.83–7.64)	0.170
Weight change in last 6 months	12/30 (40.0)	1/30 (3.3)	19.33 (2.31–161.57)	0.001
Family relationship quality not rated as good	11/30 (36.7)	3/30 (10.0)	5.21 (1.28–21.24)	0.030

ORs are unadjusted and are presented for descriptive interpretation. For each indicator, the reference category is absence of the indicator. Non-normal eating behavior was defined as eating less, overeating, or selective eating rather than normal eating. CIs were calculated using the log odds-ratio approximation; *p*-values are Fisher exact tests for the binary contrast. Because of the small sample and sparse cells, estimates should be interpreted cautiously. Undernutrition, moderate anthropometric undernutrition; OR, odds ratio; CI, confidence interval.

### Child behavioral and emotional profiles

CBCL analyses were conducted separately for the preschool and school-age assessment groups.

Among preschool-aged children (undernutrition, *n* = 9; controls, *n* = 8), no statistically significant group differences were detected on CBCL/1.5-5 syndrome scales, broadband internalizing or externalizing scores, or total problems (all *p* > 0.05; Table 3).

**Table 3:** CBCL/1.5-5 scores by group.

CBCL/1.5-5 scale	Undernutrition mean ± SD	Undernutrition median (min-max)	Control mean ± SD	Control median (min-max)	*p*
Withdrawn	3.5 ± 3.3	3.0 (0–11)	5.0 ± 5.1	4.0 (1–17)	0.481
Somatic Complaints	1.4 ± 1.4	1.0 (0–4)	3.0 ± 2.8	2.5 (0–9)	0.236
Anxiety/Depression	7.3 ± 3.6	6.0 (3–12)	6.2 ± 5.6	5.0 (1–19)	0.370
Sleep Problems	2.3 ± 1.8	2.0 (0–6)	3.6 ± 2.6	3.0 (0–8)	0.277
Attention Problems	5.6 ± 3.7	7.0 (0–12)	5.2 ± 5.4	3.5 (0–17)	0.606
Aggressive Behavior	9.7 ± 6.5	9.0 (2–22)	9.8 ± 10.3	7.5 (1–33)	0.743
Internalizing Problems	12.3 ± 7.7	12.0 (4–26)	14.2 ± 13.1	11.0 (3–45)	0.963
Externalizing Problems	15.4 ± 9.1	17.0 (2–29)	15.1 ± 15.6	12.5 (2–50)	0.606
Total Problems	30.1 ± 16.5	26.0 (8–58)	33.0 ± 30.9	28.5 (7–103)	0.673

Data are presented as mean ± SD and median (min-max). Group sizes were Undernutrition *n* = 9 and control *n* = 8.

In the CBCL/6-18 assessment band (undernutrition, *n* = 21; controls, *n* = 22), primary analyses used age- and sex-normed *T*-scores (Table 4). The undernutrition group had higher Somatic Complaints (*p* = 0.002), Anxious/Depressed (*p* = 0.015), Social Problems (*p* = 0.038), Attention Problems (*p* = 0.007), Internalizing Problems (*p* = 0.017), and Total Problems (*p* = 0.032) *T*-scores. Withdrawn/Depressed, Rule-Breaking Behavior, Aggressive Behavior, Externalizing Problems, and Thought Problems did not differ significantly (all *p* > 0.05). Hedges' g estimates for the six nominally significant domains were Somatic Complaints, 0.98 (95% CI: 0.35 to 1.60); Internalizing Problems, 0.84 (0.22 to 1.45); Total Problems, 0.75 (0.14 to 1.36); Anxious/Depressed, 0.72 (0.11 to 1.32); Social Problems, 0.71 (0.10 to 1.31); and Attention Problems, 0.57 (−0.03 to 1.17). The wide CIs indicate limited precision and are not adjusted for multiplicity. The observed age range was 6–15 years. The groups did not differ significantly in age (9.19 ± 2.46 vs. 9.55 ± 3.31 years, *p* = 0.806) or sex distribution (girls: 52.4% vs. 59.1%, Fisher's exact *p* = 0.763), but these non-significant comparisons do not establish equivalence. The same six-domain nominal pattern was present in the raw-score, age- and sex-adjusted, and rank-based sensitivity analyses (Supplementary Table S1), indicating that the principal pattern was preserved after normative standardization. After Benjamini-Hochberg correction across the 11 *T*-score comparisons, Somatic Complaints (*q* = 0.020), Attention Problems (*q* = 0.036), Anxious/Depressed (*q* = 0.047), and Internalizing Problems (*q* = 0.047) remained significant. Social Problems and Total Problems (both *q* = 0.071) did not meet the FDR threshold and were retained as preliminary components of the six-domain pattern.

**Table 4:** Age- and sex-normed CBCL/6-18 *T*-scores by group.

CBCL/6–18 *T*-score	Undernutrition mean ± SD	Undernutrition median (min-max)	Control mean ± SD	Control median (min-max)	*p*	Hedges’ g (95% CI)
Withdrawn/Depressed	55.5 ± 5.1	54.0 (50–63)	55.8 ± 6.6	54.0 (50–77)	0.901	−0.04 (−0.63 to 0.55)
Somatic Complaints	59.1 ± 7.0	57.0 (50–70)	53.3 ± 4.4	50.5 (50–64)	0.002	0.98 (0.35 to 1.60)
Anxious/Depressed	58.1 ± 8.7	54.0 (50–76)	52.8 ± 5.5	50.0 (50–72)	0.015	0.72 (0.11 to 1.32)
Internalizing Problems	57.5 ± 8.6	57.0 (45–73)	50.0 ± 8.9	50.0 (33–66)	0.017	0.84 (0.22 to 1.45)
Rule-Breaking Behavior	55.0 ± 5.0	55.0 (50–67)	52.7 ± 4.0	50.0 (50–63)	0.112	0.50 (−0.10 to 1.09)
Aggressive Behavior	54.9 ± 8.4	51.0 (50–84)	52.2 ± 3.7	50.0 (50–61)	0.278	0.41 (−0.19 to 1.00)
Externalizing Problems	50.5 ± 11.2	51.0 (33–75)	46.9 ± 8.4	46.0 (34–60)	0.324	0.36 (−0.24 to 0.95)
Social Problems	58.9 ± 7.4	57.0 (50–83)	54.4 ± 5.0	51.0 (50–64)	0.038	0.71 (0.10 to 1.31)
Thought Problems	54.2 ± 5.7	51.0 (50–67)	51.5 ± 2.3	50.0 (50–58)	0.249	0.61 (0.01 to 1.21)
Attention Problems	55.3 ± 6.7	53.0 (50–79)	52.1 ± 3.7	50.5 (50–66)	0.007	0.57 (−0.03 to 1.17)
Total Problems	50.9 ± 9.2	53.0 (36–73)	43.9 ± 9.0	44.0 (25–61)	0.032	0.75 (0.14 to 1.36)

Data are age- and sex-normed *T*-scores presented as mean ± SD and median (min-max). Group sizes were Undernutrition *n* = 21 and control *n* = 22. *P*-values are from two-sided Mann–Whitney U tests. Hedges’ g is the small-sample-corrected standardized mean difference based on *T*-scores; positive values indicate higher scores in the Undernutrition group. Ninety-five percent CIs were obtained using a noncentral-t-based method and were not adjusted for multiplicity. The Mann–Whitney U *p*-values and the Hedges’ g confidence intervals arise from different analytic frameworks and therefore need not yield identical inferential conclusions.

### Parent-proxy quality of life, maternal psychological distress, and parenting-related emotion regulation

Parent-proxy PedsQL scores are summarized in Table 5. No statistically significant group differences were detected for the PedsQL Total Score, Physical Health Summary, Psychosocial Health Summary, Emotional Functioning, Social Functioning, School Functioning, or Physical Functioning domains (all *p* > 0.05).

**Table 5:** PedsQL parent-proxy scores by group.

PedsQL scale	Undernutrition mean ± SD	Undernutrition median (min-max)	Control mean ± SD	Control median (min-max)	*p*
Physical Functioning	78.44 ± 18.63	81.25 (15.63–100.00)	78.54 ± 15.76	79.69 (46.88–100.00)	0.801
Emotional Functioning	82.50 ± 17.70	85.00 (40.00–100.00)	87.33 ± 13.11	90.00 (50.00–100.00)	0.384
Social Functioning	86.50 ± 20.68	97.50 (10.00–100.00)	88.17 ± 15.95	97.50 (45.00–100.00)	0.937
School Functioning	82.60 ± 18.49	85.00 (30.00–100.00)	83.77 ± 16.18	90.00 (25.00–100.00)	0.993
Physical Health Summary	78.76 ± 18.88	81.25 (15.63–100.00)	78.54 ± 15.76	79.69 (46.88–100.00)	0.761
Psychosocial Health Summary	84.30 ± 15.53	92.08 (28.00–100.00)	86.21 ± 10.03	85.77 (65.00–100.00)	0.988
Total Score	82.07 ± 14.00	85.99 (41.30–100.00)	83.47 ± 10.85	83.70 (58.70–100.00)	0.912

Scores range from 0 to 100, with higher scores indicating better health-related quality of life. Data are presented as mean ± SD and median (min-max).

Maternal BSI scores are shown in Table 6. The undernutrition and control groups did not differ significantly on the BSI Global Severity Index, Severe Disturbance Index, or any symptom subscale, including somatization, depression, anxiety, hostility, and negative self-concept (all *p* > 0.05). Maternal PERS scores are shown in Table 7. No significant group differences were observed in orientation to the child's emotions, avoidance of the child's emotions, acceptance of the child's and parent's own emotions, or PERS total score (all *p* > 0.05).

**Table 6:** Maternal BSI scores by group.

BSI scale/index	Undernutrition mean ± SD	Undernutrition median (min-max)	Control mean ± SD	Control median (min-max)	*p*
Somatization	0.629 ± 0.712	0.388 (0–2.888)	0.526 ± 0.509	0.444 (0–2.333)	0.976
Depression	0.675 ± 0.789	0.458 (0–3.666)	0.777 ± 0.859	0.458 (0–3.583)	0.689
Anxiety	0.450 ± 0.758	0.230 (0–3.846)	0.312 ± 0.380	0.230 (0–1.846)	0.732
Hostility	0.690 ± 0.691	0.643 (0–2.857)	0.542 ± 0.508	0.428 (0–2.000)	0.502
Negative Self-Concept	0.378 ± 0.554	0.333 (0–2.666)	0.330 ± 0.472	0.166 (0–2.000)	0.746
Severe Disturbance Index	0.053 ± 0.060	0.033 (0–0.292)	0.043 ± 0.042	0.031 (0–0.189)	0.790
Global Severity Index	0.543 ± 0.641	0.358 (0–3.188)	0.465 ± 0.448	0.310 (0–2.018)	0.859

Data are presented as mean ± SD and median (min-max). Higher values indicate greater severity of psychological symptoms.

**Table 7:** Maternal PERS scores by group.

PERS scale	Undernutrition mean ± SD	Undernutrition median (min-max)	Control mean ± SD	Control median (min-max)	*p*
Orientation to the child's emotions	24.13 ± 4.77	24.50 (13–30)	24.83 ± 5.53	26.50 (6–30)	0.365
Avoidance of the child's emotions	15.43 ± 2.78	15.00 (7–19)	15.07 ± 2.84	15.50 (6–20)	0.626
Acceptance of the child's/own emotions	10.27 ± 3.44	11.50 (3–15)	10.93 ± 3.40	12.00 (3–15)	0.503
Total score	49.70 ± 10.10	52.50 (23–62)	49.83 ± 11.23	54.50 (15–63)	0.651

Data are presented as mean ± SD and median (min-max). Higher values indicate greater endorsement of the corresponding parenting-related emotion regulation dimensions.

### Within-group correlational analyses

Within-group correlations are presented in Table 8. CBCL/6-18 correlations used age- and sex-normed *T*-scores. In the undernutrition group, Internalizing Problems *T*-scores were negatively correlated with PedsQL Total (*r* = −0.458, *p* = 0.037) and Psychosocial Health Summary scores (*r* = −0.543, *p* = 0.011). Externalizing Problems *T*-scores were also negatively correlated with PedsQL Total (*r* = −0.633, *p* = 0.002) and Psychosocial scores (*r* = −0.599, *p* = 0.004). Comparable associations were not detected in controls. After Benjamini-Hochberg correction across the 16 primary within-group correlations, the undernutrition-group Internalizing-Psychosocial association (*q* = 0.035), both Externalizing associations (both *q* = 0.033), and the two control-group maternal BSI associations (both *q* = 0.035) remained significant; the Internalizing-Total association did not (*q* = 0.084). In the all-Spearman sensitivity analysis, the directions were preserved but attenuated: undernutrition-group Internalizing-PedsQL Total, rho = −0.407, *p* = 0.067, *q* = 0.126; Internalizing-Psychosocial, rho = −0.542, *p* = 0.011, *q* = 0.060; Externalizing-PedsQL Total, rho = −0.438, *p* = 0.047, *q* = 0.125; and Externalizing-Psychosocial, rho = −0.506, *p* = 0.019, *q* = 0.062. No Spearman correlation met *q* < 0.05 (Supplementary Table S3).

**Table 8:** Correlations between key study variables by group.

Group (*n*)	Predictor variable	PedsQL total, coefficient (*p*)	PedsQL Psychosocial, coefficient (*p*)
Undernutrition (*n* = 21)	CBCL Internalizing Problems *T*-score	−0.458 (0.037)	−0.543 (0.011)
Undernutrition (*n* = 21)	CBCL Externalizing Problems *T*-score	−0.633 (0.002)	−0.599 (0.004)
Control (*n* = 22)	CBCL Internalizing Problems *T*-score	−0.255 (0.253)	−0.136 (0.545)
Control (*n* = 22)	CBCL Externalizing Problems *T*-score	−0.252 (0.258)	−0.167 (0.457)
Undernutrition (*n* = 30)	Maternal BSI Global Severity Index	0.081 (0.669)	0.107 (0.572)
Control (*n* = 30)	Maternal BSI Global Severity Index	−0.469 (0.009)	−0.479 (0.007)
Undernutrition (*n* = 30)	Maternal PERS total score	−0.354 (0.055)	−0.334 (0.071)
Control (*n* = 30)	Maternal PERS total score	−0.427 (0.019)	−0.293 (0.116)

Coefficients and *p*-values are shown. CBCL-PedsQL correlations used age- and sex-normed CBCL/6-18 *T*-scores and Pearson r; maternal BSI and PERS correlations used Spearman rho. CBCL correlations were computed in the CBCL/6-18 subsample; maternal correlations used the full sample. Undernutrition, moderate anthropometric undernutrition; CBCL, child behavior checklist; PedsQL, pediatric quality of life inventory; BSI, brief symptom inventory; PERS, parent emotion regulation ccale.

Maternal distress showed a different pattern. In controls, higher BSI Global Severity Index was associated with lower child PedsQL Total and Psychosocial scores (rho = −0.469, *p* = 0.009; rho = −0.479, *p* = 0.007), whereas these associations were not significant in the undernutrition group. Parenting-related emotion regulation showed negative correlations with child quality of life: higher PERS total scores were correlated with lower PedsQL Total scores in the undernutrition group (rho = −0.354, *p* = 0.055) and controls (rho = −0.427, *p* = 0.019). The PERS-PedsQL Psychosocial correlations were rho = −0.334 (*p* = 0.071) in the undernutrition group and rho = −0.293 (*p* = 0.116) in controls. In the primary 16-correlation family, the two control-group BSI associations remained significant after correction (both *q* = 0.035); none of the PERS correlations met *q* < 0.05.

## Discussion

### Principal findings

The present study compared behavioral and emotional functioning, parent-proxy health-related quality of life, maternal psychological distress, and parenting-related emotion regulation between children with moderate anthropometric undernutrition and healthy controls. No significant between-group differences were detected in the preschool CBCL subgroup. Primary age- and sex-normed CBCL/6-18 *T*-score analyses identified higher somatic complaints, anxious/depressed symptoms, social problems, attention problems, internalizing problems, and total problems in the undernutrition group, whereas externalizing problems were not elevated. This nominal six-domain pattern was the same as that observed in the raw-score and covariate-adjusted sensitivity analyses. Somatic Complaints, Attention Problems, Anxious/Depressed, and Internalizing Problems remained significant after FDR correction; Social Problems and Total Problems are retained as preliminary, hypothesis-generating components of the broader pattern. No significant between-group differences were found in parent-proxy quality of life, maternal psychological distress, or parenting-related emotion regulation.

Taken together, these findings describe a pattern in which outpatient moderate anthropometric undernutrition co-occurred with selected school-age somatic, internalizing, attentional, and social difficulties. This pattern may become more visible as academic, attentional, and peer-related demands intensify. Previous studies have reported associations between childhood malnutrition and school-age cognitive, emotional, attentional, and social outcomes, but many involved severe, early-life, or hospital-based malnutrition and cannot be directly generalized to the present outpatient group (12, 29–32). The current findings therefore indicate co-occurring psychosocial screening signals rather than consequences attributable to undernutrition. At the same time, the absence of broad group-level differences in parent-proxy quality of life, maternal psychological distress, and parenting-related emotion regulation indicates that the observed profile was selective rather than generalized.

### Age-specific pattern and selective symptom profile

The contrast between the preschool and school-age assessment bands is one of the most informative findings. No significant behavioral or emotional differences were detected in preschool children. This should not be interpreted as evidence that younger children with moderate anthropometric undernutrition are unaffected, because the preschool subgroup was small and subtle regulatory or socio-emotional difficulties may have been missed. Rather, the present data indicate that caregiver-reported group differences were not detectable before school age in this sample.

In school-age children, moderate anthropometric undernutrition was associated with a clearer pattern of internalizing, somatic, attentional, and social difficulties. This age-specific pattern may reflect the greater visibility of internalizing, attentional, and social difficulties when school entry increases academic, attentional, and peer-related demands. Sustained attention, emotional self-regulation, classroom participation, peer negotiation, and tolerance of bodily discomfort become more visible at school age, when nutritional vulnerability may also intersect with broader psychosocial and adolescent developmental trajectories (30–32). Given the cross-sectional design and small age-stratified subgroups, this age-related pattern should be understood as a descriptive age-band difference rather than as evidence of delayed causal emergence.

The symptom profile was selective rather than global. The use of age- and sex-normed *T*-scores directly addressed developmental and sex-related differences in CBCL score interpretation, and the same six-domain nominal pattern was reproduced in raw-score and covariate-adjusted sensitivity analyses. Nevertheless, the effect-size CIs remained wide and were not adjusted for multiplicity. Somatic Complaints, Attention Problems, Anxious/Depressed, and Internalizing Problems survived FDR correction; Social Problems and Total Problems should therefore be regarded as preliminary signals whose magnitude and reproducibility require confirmation. This distribution supports a cautious internalizing-attentional and somatic screening interpretation rather than broad behavioral dysregulation. Evidence from starvation contexts also indicates that energy insufficiency can be accompanied by psychological and cognitive changes, although such literature involves distinct clinical populations and should not be directly equated with primary-care moderate anthropometric undernutrition (33).

Attention and social problems may also be related. Attentional control and self-regulation are central to classroom functioning and peer interaction. The absence of elevated withdrawn/depressed scores suggests that the social signal may not primarily reflect social withdrawal, but may instead involve social competence, flexibility, cue monitoring, or response selection. Neurodevelopmental frameworks provide a plausible context for these attention findings because nutrition has been linked in previous literature to brain development, executive-control systems, neurophysiological trajectories, and possible low-cost biomarkers of long-term protein-energy malnutrition (30, 34–36). Broader life-course models of biological embedding also offer a framework for viewing nutritional adversity as one possible component of stress-regulatory and developmental risk (37–39). Recent neurophysiological work has reported slower nerve conduction in malnourished young children and improvement after nutritional rehabilitation (40). These findings arose from distinct populations and do not establish a neurological mechanism for the associations observed in the present study. Because cognition, nerve conduction, biomarkers, EEG, and neuroimaging were not assessed here, the attention-problem finding should be interpreted as a behavioral screening signal.

Somatic complaints may reflect bodily expressions of distress, nutrition-related physical vulnerability, or increased caregiver attention to bodily symptoms during active outpatient follow-up. In children monitored for eating, weight gain, growth, and digestion, caregivers and children may become more attentive to bodily sensations. The present design cannot distinguish these possibilities. However, recurrent somatic complaints during undernutrition follow-up may warrant psychosocial inquiry rather than being interpreted only as routine physical complaints. This interpretation is compatible with developmental approaches to early socio-emotional assessment and with evidence that somatization can be shaped by cultural and self-construal factors in Turkish clinical samples (41, 42).

Externalizing Problems were not elevated at the group level, which argues against a generalized psychopathology interpretation. However, within the undernutrition group, higher Externalizing Problems *T*-scores were associated with lower parent-proxy total and psychosocial quality-of-life scores in the primary Pearson analyses, and both associations survived the corresponding FDR correction. Their directions were preserved but their magnitudes were attenuated in the all-Spearman sensitivity analysis, in which neither survived separate FDR correction. Externalizing symptoms may therefore be functionally relevant when present, but the strength of this symptom-function relationship is sensitive to the correlation metric and requires cautious interpretation.

### Feeding behavior, quality of life, and family context

Feeding behavior provides an important family-context signal. Children with moderate anthropometric undernutrition were more often described as eating less or showing selective eating, whereas normal eating behavior was more common in controls. Reported weight change during the previous 6 months was also more frequent in the undernutrition group, suggesting a more active or unstable recent nutritional course rather than a static anthropometric classification. Mothers in the undernutrition group were also less likely to rate family relationship quality as good. These findings suggest that outpatient moderate anthropometric undernutrition may be embedded in everyday feeding routines, recent weight-related concerns, and family interactions, not merely in anthropometric status. The odds-ratio summary supported the direction of these categorical differences, but the wide CIs reinforce the exploratory nature of these estimates. The broader picky-eating literature shows that selective eating is a heterogeneous phenotype associated with sensory sensitivity, anxiety, rigidity, weight or dietary patterns, and multifactorial developmental antecedents (43–48). Caregiver stress and anxiety screening is also increasingly emphasized in pediatric feeding-disorder contexts (49). However, because feeding behavior, recent weight change, and family relationship quality were assessed descriptively rather than through validated feeding-disorder, dietary-intake, or family-functioning instruments, they should be interpreted as contextual clinical indicators rather than evidence of a specific causal family mechanism.

Mean parent-proxy quality-of-life scores did not differ between groups. However, the within-group *T*-score correlation pattern suggests a more nuanced interpretation. In the undernutrition group, higher Internalizing Problems *T*-scores were associated with lower total and psychosocial quality-of-life scores, and higher Externalizing Problems *T*-scores showed similar negative associations. The Internalizing-Psychosocial and both Externalizing associations survived FDR correction in the primary Pearson family, whereas none survived the separate all-Spearman correction. Comparable CBCL-PedsQL associations were not observed in controls. Thus, group-level quality-of-life means may mask symptom-linked functional vulnerability, but the metric sensitivity of these correlations requires caution (23, 50–52).

This symptom-function coupling is central to the quiet-burden interpretation. A child with moderate anthropometric undernutrition may appear broadly comparable to peers on global parent-proxy quality-of-life ratings while still showing internalizing, somatic, attentional, or social difficulties that relate to everyday functioning. The absence of a mean PedsQL difference therefore should not be read as evidence that psychosocial functioning is unaffected.

Maternal psychological distress did not differ between groups at the mean level. This finding may appear unexpected because previous studies in severe acute malnutrition and malnutrition-clinic contexts have linked maternal depression or distress with child malnutrition (53–55). However, meta-analytic evidence indicates that associations between maternal depression and early childhood growth outcomes are context-dependent and heterogeneous (56). In the present sample, the absence of group differences may reflect the moderate outpatient profile, the matched primary-care context, limited power, shared background adversity across groups, or the possibility that a global symptom inventory did not capture caregiver strain related specifically to feeding difficulty, growth monitoring, worry about weight gain, or mealtime tension.

The correlation between maternal distress and child quality of life adds further nuance. In controls, higher maternal distress was associated with lower child quality-of-life ratings, whereas this association was not observed in the undernutrition group. One possible interpretation is that caregivers of children under active nutritional follow-up may anchor quality-of-life judgments more strongly to physical recovery, feeding, and growth-related concerns than to their own emotional distress. Small sample size, restricted variability, and shared-informant effects may also have influenced this pattern. In addition, contextual adversity, caregiver psychological distress, and maternal self-stigma or guilt may shape how caregiver burden is expressed and reported in malnutrition contexts (57–59). This apparent decoupling should therefore be treated as an exploratory observation rather than as a confirmed psychological mechanism. The broader intervention literature nevertheless supports attention to maternal well-being as a potentially relevant component of child-related outcomes in low- and middle-income contexts (60).

Parenting-related emotion regulation showed negative directional correlations with child quality-of-life ratings, but these correlations did not remain significant after FDR correction. Together with the lower caregiver-rated family relationship quality in the undernutrition group, this pattern may be viewed within a transactional developmental framework as a contextual association among child feeding difficulties, internalizing symptoms, somatic complaints, caregiver responses, and family climate, rather than as evidence of temporal directionality (13–15). Parenting-stress evidence further suggests that child behavioral profiles and caregiver strain can be reciprocally linked across development (61, 62). Longitudinal studies using validated measures of parenting stress, caregiver burden, food insecurity, mealtime interaction, and family functioning are needed to clarify whether family processes are contributors to, consequences of, or correlates of undernutrition-related psychosocial vulnerability.

### Clinical and primary-care implications

The observed pattern suggests that primary-care nutritional follow-up may reasonably include consideration of psychosocial functioning in addition to growth monitoring. Brief, low-burden behavioral screening could be considered at key developmental transitions, particularly school entry and early adolescence. Screening may include internalizing symptoms, somatic complaints, attention problems, social functioning, and externalizing symptoms, because externalizing difficulties may be functionally costly when present even if they are not elevated at the group level. These suggestions are clinical implications of an exploratory association and should not be interpreted as evidence that undernutrition causes the identified symptoms. Accordingly, routine outpatient nutritional monitoring may be interpreted alongside the child's psychosocial functioning, feeding behavior, and family context.

Feeding behavior and family stress should also be assessed when behavioral or emotional symptoms are reported. This does not mean that all children with moderate anthropometric undernutrition require specialist psychiatric referral. A stepped-care approach may be more appropriate: routine behavioral and feeding screening for all children under active undernutrition follow-up, closer psychosocial monitoring for children with elevated symptoms, and referral when symptoms impair feeding, school participation, family life, or daily functioning.

This approach should not be framed as replacing nutritional treatment. Rather, it strengthens nutritional follow-up by identifying children whose emotional, attentional, feeding-related, or family-context needs may interfere with recovery and well-being. These findings are relevant to integrated child mental-health and primary-care models because they link nutritional vulnerability with child symptoms, feeding behavior, family context, and perceived functioning.

### Directionality and residual confounding

The observed associations permit several competing explanations. Nutritional vulnerability may precede and contribute to psychosocial difficulties through biological, developmental, or contextual mechanisms. The reverse direction is also plausible: behavioral, attentional, emotional, or feeding difficulties may disrupt mealtime routines, restrict dietary variety or intake, and thereby precipitate or maintain low weight. The higher frequencies of eating less, selective eating, and recent weight change in the undernutrition group are compatible with this possibility, but they do not establish temporal ordering. A third explanation is that nutritional status and psychosocial difficulties arise from shared antecedents, such as food insecurity, family circumstances, caregiving factors, early adversity, or unmeasured health conditions. The cross-sectional data cannot distinguish among these pathways.

Residual confounding also remains possible. Age- and sex-normed CBCL/6-18 *T*-scores reduced confounding of score interpretation by these two normative factors, and raw-score sensitivity analyses adjusted for age and sex reproduced the nominal pattern. However, normative standardization does not control for socioeconomic, familial, clinical, nutritional, or other unmeasured differences. The study had limited power to detect potentially meaningful imbalance, and non-significant baseline comparisons should not be interpreted as evidence of equivalence. The findings therefore describe co-occurrence among children receiving primary-care nutritional follow-up rather than evidence that undernutrition produces psychosocial difficulties.

### Strengths, limitations, and future directions

The main strength of this study is its focus on moderate anthropometric undernutrition in an outpatient primary-care follow-up context, a group that is clinically common but less often characterized in the child mental-health literature. The study used developmentally appropriate CBCL forms, assessed parent-proxy quality of life, included maternal psychological and parenting-related emotion-regulation measures, and examined symptom-function associations within groups rather than relying only on mean group comparisons. The inclusion of feeding behavior and family relationship indicators also allowed a broader clinical interpretation.

Several limitations should be acknowledged. The cross-sectional design cannot establish whether moderate anthropometric undernutrition contributes to, results from, or merely co-occurs with the observed psychosocial profile. No temporal ordering can be inferred among nutritional status, behavioral and emotional symptoms, feeding behavior, quality-of-life ratings, and family characteristics. The study was not designed to test bidirectional transactional developmental mechanisms; the transactional framework was used only as an interpretive context. The exact diagnosis-to-assessment interval was not analyzed, which further limits conclusions about temporal stage within outpatient nutritional follow-up. The sample was small, particularly in the preschool subgroup, limiting statistical power and increasing the possibility of missed associations. All child and family measures were completed by mothers, creating potential shared-informant variance. Parent-proxy reports are clinically useful but cannot replace teacher reports, child self-report when age-appropriate, clinician-rated psychiatric assessment, or structured diagnostic interviews (50–52). Children in the undernutrition group also had greater healthcare contact, which may have increased caregiver attention to bodily symptoms and contributed to higher somatic-complaint ratings.

Methodological limitations further require caution. Although licensed age- and sex-normed CBCL/6-18 *T*-scores were used for the primary analyses, normative scoring does not eliminate confounding beyond age and sex, and the absence of statistically significant baseline differences should not be interpreted as evidence of equivalence. Residual confounding by measured and unmeasured sociodemographic, familial, clinical, and nutritional factors remains possible. Benjamini-Hochberg correction was applied within conceptually coherent test families. Within the CBCL/6-18 *T*-score family, Somatic Complaints, Attention Problems, Anxious/Depressed, and Internalizing Problems survived correction, whereas Social Problems and Total Problems did not and should be interpreted as preliminary. Effect-size estimates were based on subgroups of 21 and 22 children and had wide CIs; they should inform replication and sample-size planning rather than be treated as stable magnitudes. The CBCL-PedsQL findings were also sensitive to the association metric: several primary Pearson associations survived FDR correction, whereas none of the all-Spearman correlations did. These within-group associations should therefore remain exploratory and may be influenced by distributional features or influential observations. The added OR estimates for categorical indicators were unadjusted, based on small cell counts, and intended only for descriptive interpretation. Nutritional management was not analyzed as a comparative exposure because it formed part of the undernutrition case definition, and caregiver-reported supplementation could not be isolated as a treatment variable. The undernutrition group was assessed while still meeting moderate anthropometric criteria, so the results may not generalize to children who have recovered. Because the group was restricted to −3 SD ≤ *z*-score < −2 SD, the findings should not be generalized to children only at risk of undernutrition (−2 SD ≤ *z*-score < −1 SD).

Finally, the study did not include dietary intake measures, micronutrient assays, biomarkers, neuropsychological testing, nerve-conduction studies, teacher reports, child self-report, validated feeding-disorder assessment, observational family measures, EEG, or neuroimaging. Future longitudinal studies should follow children from undernutrition diagnosis through nutritional rehabilitation and school transition. Multi-informant designs are especially important: teacher reports may clarify attention and peer-functioning difficulties, while child self-report may better capture internalizing symptoms in older children and adolescents. Larger samples should also examine modifiable risk and protective factors, including food insecurity, caregiver burden, parenting stress, mealtime interaction, family functioning, micronutrient status, school support, and access to primary mental-health services.

Future intervention studies should test whether integrated nutritional and psychosocial follow-up improves both anthropometric and functional outcomes. Existing cognitive and neurophysiological evidence supports the developmental relevance of nutritional vulnerability, but it does not establish which combined nutritional-psychosocial intervention components are effective for children with outpatient moderate anthropometric undernutrition (12, 40). Community-based nutrition-support models may also be relevant to biological rehabilitation, but their local feasibility, cultural acceptability, and psychosocial effects should be evaluated before generalization (63).

## Conclusion

In conclusion, primary age- and sex-normed CBCL/6-18 *T*-score analyses identified a selective school-age psychosocial pattern associated with outpatient moderate anthropometric undernutrition. Somatic Complaints, Attention Problems, Anxious/Depressed, and Internalizing Problems were robust to FDR correction, whereas the Social Problems and Total Problems findings remain hypothesis-generating. Group-level quality of life and maternal psychological distress appeared preserved, but *T*-score-based symptom-function associations within the undernutrition group suggest potentially meaningful functional vulnerability and require cautious interpretation because of metric sensitivity. These findings support consideration of brief behavioral screening, feeding-behavior assessment, and family-sensitive support during primary-care nutritional follow-up. They do not establish that undernutrition causes the observed psychosocial profile. Longitudinal, multi-informant studies are needed to clarify directionality, shared determinants, and whether anthropometric and psychosocial changes track one another over time.

## Data Availability

The datasets generated and/or analyzed during the current study are not publicly available due to ethical and privacy restrictions but are available from the corresponding author upon reasonable request.
