# Supplementary material for: School-age somatic, internalizing, attention, and social difficulties in moderate anthropometric undernutrition: a primary-care case-control study
Source: Front Pediatr. 2026 Aug 18;14:1893524. doi: 10.3389/fped.2026.1893524 (PMC13529972; doi:10.3389/fped.2026.1893524)
Supplement: Supplementary file 1 [file Supplementaryfile1.docx]

**Supplementary Material**

*School-age somatic, internalizing, attention, and social difficulties in moderate anthropometric undernutrition: a primary-care case-control study*

**Supplementary Table S1. Raw-score and age- and sex-adjusted sensitivity analyses for CBCL/6-18 outcomes**

| CBCL/6-18 scale | Raw-score unadjusted p | Age- and sex-adjusted p | Rank-ANCOVA p | Raw-score Hedges' g (95% CI) | Residualized Hedges’ g (95% CI) |
| --- | --- | --- | --- | --- | --- |
| Withdrawn/Depressed | 0.980 | 0.763 | 0.917 | -0.12 (-0.71 to 0.47) | -0.09 (-0.68 to 0.50) |
| Somatic Complaints | 0.002 | 0.001 | 0.001 | 1.01 (0.38 to 1.63) | 1.05 (0.41 to 1.67) |
| Anxious/Depressed | 0.039 | 0.029 | 0.042 | 0.67 (0.06 to 1.27) | 0.69 (0.08 to 1.29) |
| Internalizing Problems | 0.019 | 0.016 | 0.014 | 0.74 (0.12 to 1.34) | 0.77 (0.15 to 1.37) |
| Rule-breaking Behavior | 0.141 | 0.170 | 0.165 | 0.44 (-0.16 to 1.03) | 0.43 (-0.17 to 1.02) |
| Aggressive Behavior | 0.364 | 0.222 | 0.380 | 0.39 (-0.20 to 0.98) | 0.38 (-0.22 to 0.97) |
| Externalizing Problems | 0.282 | 0.182 | 0.308 | 0.44 (-0.16 to 1.03) | 0.41 (-0.18 to 1.01) |
| Social Problems | 0.017 | 0.015 | 0.018 | 0.79 (0.18 to 1.40) | 0.77 (0.16 to 1.38) |
| Thought Problems | 0.254 | 0.120 | 0.325 | 0.52 (-0.08 to 1.12) | 0.49 (-0.11 to 1.08) |
| Attention Problems | 0.006 | 0.019 | 0.006 | 0.77 (0.15 to 1.37) | 0.75 (0.13 to 1.35) |
| Total Problems | 0.029 | 0.028 | 0.031 | 0.71 (0.10 to 1.31) | 0.70 (0.09 to 1.30) |

Note. Table 4 presents the primary age- and sex-normed T-score analysis. This supplementary table retains the former raw-score comparisons and reports the study-group term from ANCOVA models including age and sex, rank-ANCOVA using rank-transformed raw scores, and Hedges' g estimates based on raw and age- and sex-residualized scores. Ninety-five percent CIs were obtained using a noncentral-t-based method and were not adjusted for multiplicity. The concordant nominal six-domain pattern supports sensitivity of the result to scoring approach but does not exclude residual confounding. All p-values are two-sided and nominal.

**Supplementary Table S2. Benjamini-Hochberg false-discovery-rate sensitivity analysis**

| Test family | Outcome/comparison | p | BH q | Interpretation |
| --- | --- | --- | --- | --- |
| CBCL/6-18 | Withdrawn/Depressed | 0.901 | 0.901 | Not significant |
| CBCL/6-18 | Somatic Complaints | 0.002 | 0.020 | Survived FDR |
| CBCL/6-18 | Anxious/Depressed | 0.015 | 0.047 | Survived FDR |
| CBCL/6-18 | Internalizing Problems | 0.017 | 0.047 | Survived FDR |
| CBCL/6-18 | Rule-breaking Behavior | 0.112 | 0.176 | Not significant |
| CBCL/6-18 | Aggressive Behavior | 0.278 | 0.340 | Not significant |
| CBCL/6-18 | Externalizing Problems | 0.324 | 0.356 | Not significant |
| CBCL/6-18 | Social Problems | 0.038 | 0.071 | Nominal only / preliminary |
| CBCL/6-18 | Thought Problems | 0.249 | 0.340 | Not significant |
| CBCL/6-18 | Attention Problems | 0.007 | 0.036 | Survived FDR |
| CBCL/6-18 | Total Problems | 0.032 | 0.071 | Nominal only / preliminary |
| CBCL/1.5-5 | Withdrawn | 0.481 | 0.836 | Not significant |
| CBCL/1.5-5 | Somatic Complaints | 0.236 | 0.836 | Not significant |
| CBCL/1.5-5 | Anxiety/Depression | 0.370 | 0.836 | Not significant |
| CBCL/1.5-5 | Sleep Problems | 0.277 | 0.836 | Not significant |
| CBCL/1.5-5 | Attention Problems | 0.606 | 0.836 | Not significant |
| CBCL/1.5-5 | Aggressive Behavior | 0.743 | 0.836 | Not significant |
| CBCL/1.5-5 | Internalizing Problems | 0.963 | 0.963 | Not significant |
| CBCL/1.5-5 | Externalizing Problems | 0.606 | 0.836 | Not significant |
| CBCL/1.5-5 | Total Problems | 0.673 | 0.836 | Not significant |
| PedsQL | Physical Functioning | 0.801 | 0.993 | Not significant |
| PedsQL | Emotional Functioning | 0.384 | 0.993 | Not significant |
| PedsQL | Social Functioning | 0.937 | 0.993 | Not significant |
| PedsQL | School Functioning | 0.993 | 0.993 | Not significant |
| PedsQL | Physical Health Summary | 0.761 | 0.993 | Not significant |
| PedsQL | Psychosocial Health Summary | 0.988 | 0.993 | Not significant |
| PedsQL | Total Score | 0.912 | 0.993 | Not significant |
| BSI | Somatization | 0.976 | 0.976 | Not significant |
| BSI | Depression | 0.689 | 0.976 | Not significant |
| BSI | Anxiety | 0.732 | 0.976 | Not significant |
| BSI | Hostility | 0.502 | 0.976 | Not significant |
| BSI | Negative Self-Concept | 0.746 | 0.976 | Not significant |
| BSI | Severe Disturbance Index | 0.790 | 0.976 | Not significant |
| BSI | Global Severity Index | 0.859 | 0.976 | Not significant |
| PERS | Orientation to child emotions | 0.365 | 0.651 | Not significant |
| PERS | Avoidance of child emotions | 0.626 | 0.651 | Not significant |
| PERS | Acceptance of child/own emotions | 0.503 | 0.651 | Not significant |
| PERS | Total score | 0.651 | 0.651 | Not significant |
| Feeding/family | Non-normal eating behavior | <0.001 | <0.001 | Survived FDR |
| Feeding/family | Eats less | 0.029 | 0.038 | Survived FDR |
| Feeding/family | Selective eating | 0.170 | 0.170 | Not significant |
| Feeding/family | Weight change in previous 6 months | 0.001 | 0.003 | Survived FDR |
| Feeding/family | Family relationship quality not rated as good | 0.030 | 0.038 | Survived FDR |
| Within-group correlations | Undernutrition: Internalizing–PedsQL Total | 0.037 | 0.084 | Nominal only / preliminary |
| Within-group correlations | Undernutrition: Internalizing–PedsQL Psychosocial | 0.011 | 0.035 | Survived FDR |
| Within-group correlations | Undernutrition: Externalizing–PedsQL Total | 0.002 | 0.033 | Survived FDR |
| Within-group correlations | Undernutrition: Externalizing–PedsQL Psychosocial | 0.004 | 0.033 | Survived FDR |
| Within-group correlations | Undernutrition: BSI GSI–PedsQL Total | 0.669 | 0.669 | Not significant |
| Within-group correlations | Undernutrition: BSI GSI–PedsQL Psychosocial | 0.572 | 0.610 | Not significant |
| Within-group correlations | Undernutrition: PERS–PedsQL Total | 0.055 | 0.110 | Not significant |
| Within-group correlations | Undernutrition: PERS–PedsQL Psychosocial | 0.071 | 0.126 | Not significant |
| Within-group correlations | Control: Internalizing–PedsQL Total | 0.253 | 0.344 | Not significant |
| Within-group correlations | Control: Internalizing–PedsQL Psychosocial | 0.545 | 0.610 | Not significant |
| Within-group correlations | Control: Externalizing–PedsQL Total | 0.258 | 0.344 | Not significant |
| Within-group correlations | Control: Externalizing–PedsQL Psychosocial | 0.457 | 0.562 | Not significant |
| Within-group correlations | Control: BSI GSI–PedsQL Total | 0.009 | 0.035 | Survived FDR |
| Within-group correlations | Control: BSI GSI–PedsQL Psychosocial | 0.007 | 0.035 | Survived FDR |
| Within-group correlations | Control: PERS–PedsQL Total | 0.019 | 0.051 | Nominal only / preliminary |
| Within-group correlations | Control: PERS–PedsQL Psychosocial | 0.116 | 0.186 | Not significant |

Note. Benjamini-Hochberg correction was applied separately within each instrument- or domain-based test family. The FDR threshold was q < 0.05. CBCL/6-18 between-group and CBCL-PedsQL correlation entries use age- and sex-normed T-scores. Exact participant-level p-values were used for the CBCL/6-18, feeding/family, and correlation families; manuscript table p-values were used for the remaining families. CBCL, Child Behavior Checklist; PedsQL, Pediatric Quality of Life Inventory; BSI, Brief Symptom Inventory; GSI, Global Severity Index; PERS, Parent Emotion Regulation Scale; FDR, false discovery rate.

**Supplementary Table S3. Spearman distributional sensitivity analysis for all within-group correlations**

| **Group** | **Predictor** | **Outcome** | **n** | **Spearman rho** | **p** | **BH q** |
| --- | --- | --- | --- | --- | --- | --- |
| Undernutrition | CBCL Internalizing Problems | PedsQL Total | 21 | -0.407 | 0.067 | 0.126 |
| Undernutrition | CBCL Internalizing Problems | PedsQL Psychosocial | 21 | -0.542 | 0.011 | 0.060 |
| Undernutrition | CBCL Externalizing Problems | PedsQL Total | 21 | -0.438 | 0.047 | 0.125 |
| Undernutrition | CBCL Externalizing Problems | PedsQL Psychosocial | 21 | -0.506 | 0.019 | 0.062 |
| Undernutrition | Maternal BSI Global Severity Index | PedsQL Total | 30 | 0.081 | 0.669 | 0.669 |
| Undernutrition | Maternal BSI Global Severity Index | PedsQL Psychosocial | 30 | 0.107 | 0.572 | 0.654 |
| Undernutrition | Maternal PERS total score | PedsQL Total | 30 | -0.354 | 0.055 | 0.126 |
| Undernutrition | Maternal PERS total score | PedsQL Psychosocial | 30 | -0.334 | 0.071 | 0.126 |
| Control | CBCL Internalizing Problems | PedsQL Total | 22 | -0.297 | 0.179 | 0.261 |
| Control | CBCL Internalizing Problems | PedsQL Psychosocial | 22 | -0.113 | 0.618 | 0.659 |
| Control | CBCL Externalizing Problems | PedsQL Total | 22 | -0.231 | 0.302 | 0.402 |
| Control | CBCL Externalizing Problems | PedsQL Psychosocial | 22 | -0.147 | 0.514 | 0.633 |
| Control | Maternal BSI Global Severity Index | PedsQL Total | 30 | -0.469 | 0.009 | 0.060 |
| Control | Maternal BSI Global Severity Index | PedsQL Psychosocial | 30 | -0.479 | 0.007 | 0.060 |
| Control | Maternal PERS total score | PedsQL Total | 30 | -0.427 | 0.019 | 0.062 |
| Control | Maternal PERS total score | PedsQL Psychosocial | 30 | -0.293 | 0.116 | 0.186 |

*Note. Spearman rank correlations were recalculated for all 16 within-group associations as a distributional sensitivity analysis. CBCL-PedsQL rows used age- and sex-normed CBCL/6-18 T-scores. Benjamini-Hochberg correction was applied across the 16 Spearman p-values; no association met q < 0.05. CBCL, Child Behavior Checklist; PedsQL, Pediatric Quality of Life Inventory; BSI, Brief Symptom Inventory; PERS, Parent Emotion Regulation Scale; FDR, false discovery rate.*
